# Metabolomics analysis of different diameter classes of *Taxus chinensis* reveals that the resource allocation is related to carbon and nitrogen metabolism

**DOI:** 10.1186/s12870-024-05070-z

**Published:** 2024-05-09

**Authors:** Liben Pan, Yi Li, Wen Zhao, Yushu Sui, Nan Yang, Longjie Liu, Yang Liu, Zhonghua Tang, Liqiang Mu

**Affiliations:** 1 School of Forestry, Northeast Forestry University, Harbin, 150040 China; 2 Key Laboratory of Forest Plant Ecology, Ministry of Education, Northeast Forestry University, Harbin, 150040 China; 3 College of Chemistry, Chemical Engineering and Resource Utilization, Northeast Forestry University, Harbin, 150040 China; 4 Institute of Botany, Chinese Academy of Sciences, Beijing, 100093 China; 5 School of Life Sciences, Heilongjiang University, Harbin, 150080 China

**Keywords:** *Taxus chinensis*, Diameter class, Metabolomics, Carbon and nitrogen metabolism, Paclitaxel

## Abstract

*Taxus chinensis* (*Taxus cuspidata* Sieb. et Zucc.) is a traditional medicinal plant known for its anticancer substance paclitaxel, and its growth age is also an important factor affecting its medicinal value. However, how age affects the physiological and metabolic characteristics and active substances of *T. chinensis* is still unclear. In this study, carbon and nitrogen accumulation, contents of active substances and changes in primary metabolites in barks and annual leaves of *T. chinensis* of different diameter classes were investigated by using diameter classes instead of age. The results showed that leaves and barks of small diameter class (D1) had higher content of non-structural carbohydrates and C, which were effective in enhancing defense capacity, while N content was higher in medium (D2) and large diameter classes (D3). Active substances such as paclitaxel, baccatin III and cephalomannine also accumulated significantly in barks of large diameter classes. Moreover, 21 and 25 differential metabolites were identified in leaves and barks of different diameter classes, respectively. The differential metabolites were enhanced the TCA cycle and amino acid biosynthesis, accumulate metabolites such as organic acids, and promote the synthesis and accumulation of active substances such as paclitaxel in the medium and large diameter classes. These results revealed the carbon and nitrogen allocation mechanism of different diameter classes of *T. chinensis*, and its relationship with medicinal components, providing a guidance for the harvesting and utilization of wild *T. chinensis*.

## Introduction

*T. chinensis* is an evergreen woody plant of *Taxus* in Taxaceae [1]. It is primarily distributed in China, Japan, Korea and the Soviet Union, and is regarded as a valuable relict tree species from the Tertiary Period. In recent years, *T. chinensis* has garnered considerable attention due to its rich content of bioactive molecules, such as paclitaxel, flavonoids, steroids, and lignans [2]. These compounds have shown promise in cancer treatment, hypoglycemia, and antioxidant properties [3, 4]. Unfortunately, the growth rate of the *T. chinensis* population is slow, and its competitiveness and regeneration ability are low [5]. Therefore, understanding the dynamic changes in paclitaxel content in different age groups of *T. chinensis* is of great significance for the development and utilization of *T. chinensis*.

Non-structural carbohydrates (NSCs), including soluble sugars and starch, are important organic carbon components in plants [6, 7]. Meanwhile, NSCs can reflect the balance between carbon assimilation and carbon consumption during plant growth and survival [8]. Soluble sugars regulate cellular osmotic pressure, aiding in stress resistance, while starch serves as a long-term nutrient pool, and both can interconvert under specific conditions. The relationships among their contents delineate plant growth and defense strategies [9, 10]. Previous studies have shown that the accumulation of primary metabolite such as soluble sugar and starch in *T. chinensis* contributes to the active substances such as paclitaxel [11]. Therefore, detecting the dynamics of carbohydrates, especially NSCs content, in different parts of plant tissues with tree age, is crucial for evaluating and understanding tree carbon dynamics.

Carbon (C), nitrogen (N) and phosphorus (P) are essential elements in the structural composition and functional metabolism of plant cells, playing a pivotal role in regulating various physiological processes in plants [12, 13]. In addition, carbon and nitrogen metabolism are the major metabolic processes in plants, both necessitating the absorption of energy generated by photosynthesis [14]. However, there is little research on the interaction between carbon metabolism and nitrogen metabolism in *T. chinensis.* According to the growth rate hypothesis, the content of C, N and P in plants is closely related to the growth rate [15]. Fast-growing plants require more rRNA input than protein input [16], meaning that the stoichiometric ratio of rapidly growing plants slopes towards P. Fast-growing plants have higher N: C and P: C ratios and lower N: P ratios [17]. The stoichiometric characteristics of each organ can reflect the optimal growth strategy adopted by the plant to adapt to its environment [18]. Therefore, investigating the stoichiometric characteristics of nutrient elements in *T. chinensis* can provide valuable point of reference for understanding the nutrient utilization strategy during its growth.

In the long-term evolution of plants, a vast array of primary metabolites with structural diversity have been synthesized. These metabolites possess a critical function in plant growth, environmental adaptation, and efficacy [19]. Gas chromatography-mass spectrometry (GC-MS) technology is of great importance in plant metabolomics research due to its high resolution, sensitivity, reproducibility, and large library of standard metabolite spectra [20]. GC-MS allows the simultaneous measurement of a large number of chemically diverse compounds, including organic acids, amino acids, sugars, sugar alcohols, aromatic amines, and fatty acids [21]. Therefore, GC-MS is widely used to reveal the changes of metabolic fluxes in different tissue parts, growth stages and different environments of plants. Zhang et al. used GC-MS to compare the active components in three different *T. chinensis* leaves, indicating that the most diverse and abundant metabolites are found in *T. chinensis* [22]. Previous studies have examined differences in leaf metabolism in different species of *T. chinensis* [23], yet relatively few studies focused on the differential response of primary metabolites to tissue and development stages.

*T. chinensis*, as an evergreen tree species with medicinal value, has attracted extensive attention due to its active substances such as paclitaxel. Previous studies mainly focused on the content of paclitaxel in *T. chinensis* of different species and different tissue parts [24]. However, growth age also significantly influences its medicinal efficacy [25]. To investigate the effects of physiological metabolic responses on the accumulation of active substances in different ages, we collected barks and leaves of the current year from different diameter classes representing different ages and analyzed them for non-structural carbohydrates, nutrients, active components, and primary metabolites in *T. chinensis*.

## Methods

### Plant materials and growth condition

The study was conducted in 2019 at Muling *Taxus cuspidata* Sieb. et Zucc. Nature Reserve, located in Heilongjiang Province, China, at the northern end of the Changbai Mountains (130°00’ ∼ 130°28’ E, 43°49’ ∼ 44°06’ N). The reserve belongs to a temperate continental monsoon climate, with an average annual temperature of -2 °C and an average annual precipitation of 530 mm. The *T. chinensis* in the reserve are distributed throughout mixed coniferous and broad-leaved forests, with a complete age structure and good regeneration. The *T. chinensis* was identified by Prof. Liqiang Mu (Northeast Forestry University), and voucher specimens of the *T. chinensis* has been deposited in the Herbarium of the Northeast Forestry University (Deposition number: 021001001001001 ∼ 021001001001004). The *T. chinensis* used in the experiment were obtained through natural regeneration.

Three sample plots with the same forest type, soil type and elevation were established. Each plot measured 30 m×30 m, with a distance of at least 50 m between each plot. In mid-September, at approximately 10 a.m., different diameter grades of each sample were collected in an “S” shape. Leaves of the current year and barks at 1.3 m above ground were collected from the south-facing crowns of female *T. chinensis* at each sample site. Breast height diameter collected ranged from 2.6 cm to 47.4 cm and was divided into three stages of growth and development according to diameter class. Breast height diameter 2.0–16.0 cm was stage D1, 16.0–32.0 cm was D2, and 32.0–48.0 cm was D3. Portions of the samples were immediately frozen in liquid nitrogen and stored at -80 °C for GC-MS analysis. Portions of the samples were dried at 105 °C for 10 min and then at 70 °C until constant weight. The dried tissues were ground and passed through a 0.5 mm sieve, and stored for analysis of non-structural carbohydrates, carbon, nitrogen, phosphorus, and active ingredients.

### Soluble sugar and starch content

Soluble sugars and starch concentrations were determined by the anthrone method [26]. Soluble sugars were first extracted twice from 0.1 g of powder using 2 mL of 80% ethanol. The residue was decomposed with 2 mL of 9.2 mol/L HClO_4,_ and 4 mL of distilled water was added for starch determination. The concentrations of soluble sugars and starch were measured by absorbance at 625 nm using a UV visible spectrophotometer. Sugar concentration was calculated from the regression equation of glucose standard solution and starch concentration. The sum of soluble sugars and starch is referred to as total non-structural carbohydrates.

### Metabolite extraction and detection

A total of 0.1 g sample was mixed with 0.8 mL of 80% methanol solution and 5 µL 2-chloro-L-phenylalanine (0.3 mg/mL). The mixture was sonicated at 4 °C for 30 min and subsequently centrifuged at 12,000 rpm and 4 °C for 15 min. The 200 µL supernatant concentrated by rotary evaporation, and then 35 µL of 20 mg/mL methoxypyridine solution was added. The mixture was vigorously shaken for 30 s and allowed to react at 37 °C for 90 min. Then, 35 µL of N, O-Bis (trimethylsilyl) trifluoroacetamide (BSTFA) (containing 1% TMCS) was added for derivatization and the reaction was carried out at 70 °C for 60 min. After 30 min at room temperature, the material was analyzed by GC-MS. The GC–MS data were obtained using the Agilent 7890 A-5975 C (Agilent, USA) and a non-polar DB-5 capillary column (30 m × 250 μm I.D., J&W Scientific, Folsom, CA). Instrument parameters: Inlet temperature 280 °C, EI ion source temperature 230 °C, high purity helium (purity > 99.999%) as carrier gas, shunt ratio 10:1, injection volume 1.0 µL, solvent delay 5 min. Ramp-up procedure: initial temperature 70 °C, ramp-up to 200 °C at 10 °C/min, then ramp-up to 280 °C at 5 °C/min and maintain for 10 min. 

### Determination of carbon, nitrogen, and phosphorus content

An elemental analyzer (Elementar Inc., Hanau, Hessen, Germany) was used to determine the carbon and nitrogen content in the leaves and barks of *T. chinensis*. The total phosphorus content was determined using a UV spectrophotometer after digestion with H_2_SO_4_ and H_2_O_2_, according to the method of Zhang et al. [27].

### Extraction and HPLC analysis for 10-deacetylbaccatin III, baccatin III, cephalomannine and paclitaxel

A leaves and barks samples of *T. chinensis* (1 g) was mixed with 30 mL of 80% ethanol, extracted by ultrasonication for 30 min, centrifuged, and the supernatant was retained. The process was repeated 3 times and the filtrates were combined and concentrated by rotary evaporation with the addition of 1mL of methanol and an equal volume of ethyl acetate, left overnight, centrifuged and the supernatant was removed. And the obtained solution was filtered through 0.22 μm membrane for injection into HPLC.

The separation was carried out on a Zorbax SB-C18 (5 μm, 4.6 mm × 25 cm, Agilent Technologies) column at 30 °C with the flow rate maintained at 1.0 mL/min. The mobile phase consisted of acetonitrile (solvent A) and water (solvent B). The injection volume was 10 µL and the peak was observed at 227 nm. The gradient program was as follows: 0–3 min, 40% A; 3–7 min, 40–45% A; 7–11 min, 45–75% A; 11–17 min, 75 − 40% A. Sample peaks were identified based on matching retention times compared to authentic standards.

### Statistical analysis

Data were analyzed using SPSS 19.0 (SPSS, Santa Clara, CA, USA) one-way factorial analysis of variance (ANOVA). In all cases, differences were considered significant at a probability level of *P* < 0.05. Plots were made with GraphPad Prism 8.

Unsupervised Principal Component Analysis (PCA) was carried out using the statistics function prcomp within R v3.5.0 (www.r-project.org). Supervised multiple regression orthogonal partial least-squares discriminant analysis (OPLS-DA) was performed using ropls v1.19.8 in R (Thevenot, Roux, Xu, Ezan, & Junot, 2015). Differential metabolites were identified using a threshold variable importance in projection (VIP) value (VIP > 1.0) and one-way ANOVA analysis (*P*<0.05). Hierarchical cluster analysis (HCA) and the corresponding Heatmap were performed using the ClustVis online tool. Annotated metabolites were mapped to the Kyoto Encyclopedia of Genes and Genomes (KEGG) pathway database (http://www.kegg.jp/kegg/pathway.html) to determine pathway associations) [28–30]. Pathway enrichment analysis was performed on the MetaboAnalyst 5.0 (https://www.metaboanalyst.ca). Pearson correlations were used to assess the relationships between physiochemical parameters by using R software (version 4.1.2).

## Results

### Non-structural carbohydrates traits in leaves and barks of *T. chinensis* in different diameter classes

**Fig. 1:**
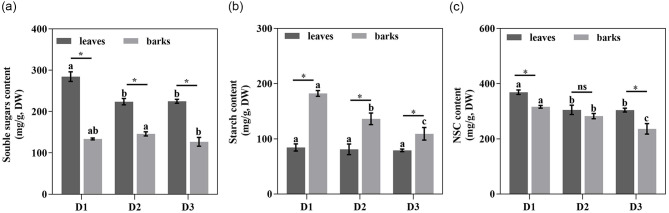
Contents of soluble sugars (**a**), starch (**b**) and non-structural carbohydrates, NSCs (**c**) in leaves and barks of *T. chinensis* in different diameter classes. Different letters indicate significant differences between different diameter classes (*P* < 0.05); * indicates significant differences (*P* < 0.05) between leaves and bark at the same diameter class

Non-structural carbohydrates, including soluble sugars and starch, are important energy substances in plants. Compared to D1, the soluble sugar content in leaves was significantly decreased in D2 and D3, by 21.32% and 20.94%, respectively. Soluble sugar content in the barks only significantly decreased in D3 (Fig. 1a). Starch content in barks decreased significantly with increasing diameter classes. Compared to D1, D2 and D3 decreased by 25.29% and 40.15%, respectively. There was no significant difference in starch content in leaves (Fig. 1b). NSCs content in both leaves and barks decreased with increasing diameter class at different diameter levels. The changes in NSCs in leaves were caused by soluble, sugars whereas changes in NSCs in barks were caused by starch (Fig. 1c).

In the same diameter class, soluble sugars accumulated mainly in leaves, while starch accumulated mainly in barks. Soluble sugar content was significantly higher in leaves than in barks, with 2.13, 1.53 and 1.77 times higher than in barks with increasing diameter, respectively. In contrast, the starch content in barks was 2.16, 1.68 and 1.38 times higher than in D1, D2 and D3 leaves, respectively. The NSCs content in both D1 and D3 leaves was significantly higher than in the barks, 1.17 and 1.29 times higher than in the barks, respectively.

### Carbon, nitrogen, and phosphorus characteristics in different diameter classes in leaves and barks of *T. chinensis*

As shown in Table 1, there was no significant effect of diameter class on C content in annual leaves. However, C content in barks decreased significantly with increasing diameter class, with a 14.67% and 16.79% decrease in D2 and D3, respectively, compared to D1. N content in leaves and barks increased with increasing diameter class. The P content in leaves increased significantly with increasing diameter class, while the P content in barks did not differ significantly among different diameter classes. In addition, the stoichiometric ratios were significantly different among the different diameter classes. The stoichiometric ratios in leaves and barks showed the same pattern of change at different diameter classes. The C: N ratio decreased with increasing diameter class. In contrast, the N: P ratio increased with increasing diameter class.

In addition, in the same diameter class, the content of C, N, and P in leaves is significantly higher than that in barks. Compared with leaves, the average C, N, and P content of the three diameter classes in the barks decreased by 14.77%, 45.03%, and 58.14%, respectively. Conversely, the stoichiometric ratios were higher in barks than in leaves for C: N, N: P and C: P ratios, and most of them were significantly different.

**Table 1:** C, N and P contents and stoichiometric ratios of *T. chinensis*

Variable	Leaves			Barks		
	D1	D2	D3	D1	D2	D3
**C (g/kg)**	588.32 ± 13.76a*	566.82 ± 20.16a*	578.43 ± 4.28a*	550.22 ± 4.24a*	469.55 ± 4.89b*	457.83 ± 15.09b*
**N (g/kg)**	9.74 ± 0.12c*	12.93 ± 0.46b*	16.89 ± 0.17a*	6.81 ± 0.41b*	7.05 ± 0.12ab*	7.89 ± 0.12a*
**P (g/kg)**	4.97 ± 0.23b*	5.82 ± 0.1a*	5.99 ± 0.31a*	2.49 ± 0.21a*	2.44 ± 0.07a*	2.11 ± 0.08a*
**C: N ratio**	59.49 ± 0.25a	43.26 ± 1.06b*	35.26 ± 0.26c*	81.38 ± 5.24a	66.67 ± 1.27b*	58.12 ± 2.62b*
**N: P ratio**	1.97 ± 0.11b	2.22 ± 0.11b*	2.84 ± 0.15a*	2.80 ± 0.42b	2.89 ± 0.08ab*	3.75 ± 0.15a*
**C: P ratio**	117.07 ± 5.83a*	96.07 ± 3.59b*	100.02 ± 6.11ab*	223.73 ± 18.43a*	192.36 ± 3.37a*	216.82 ± 5.24a*

### Active substances accumulation in different diameter classes in leaves and barks of *T. chinensis*

**Fig. 2:**
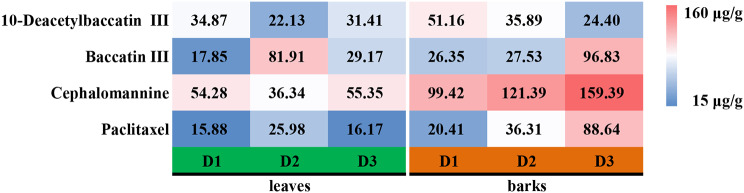
The content of active substances in leaves and barks of different diameter classes of *T. chinensis*

To further compare the differences in active substances in different diameter classes of T. chinensis, the contents of 10-deacetylbaccatin III, baccatin III, cephalomannine and paclitaxel were determined by HPLC. When the diameter classes were consistent, most active substances were higher in barks than in leaves. Except for 10-deacetylbaccatin III, the content of other active substances in barks increased significantly with increasing diameter class (Fig. 2).

### Metabolic profiling of *T. chinensis* based on GC-MS

**Fig. 3:**
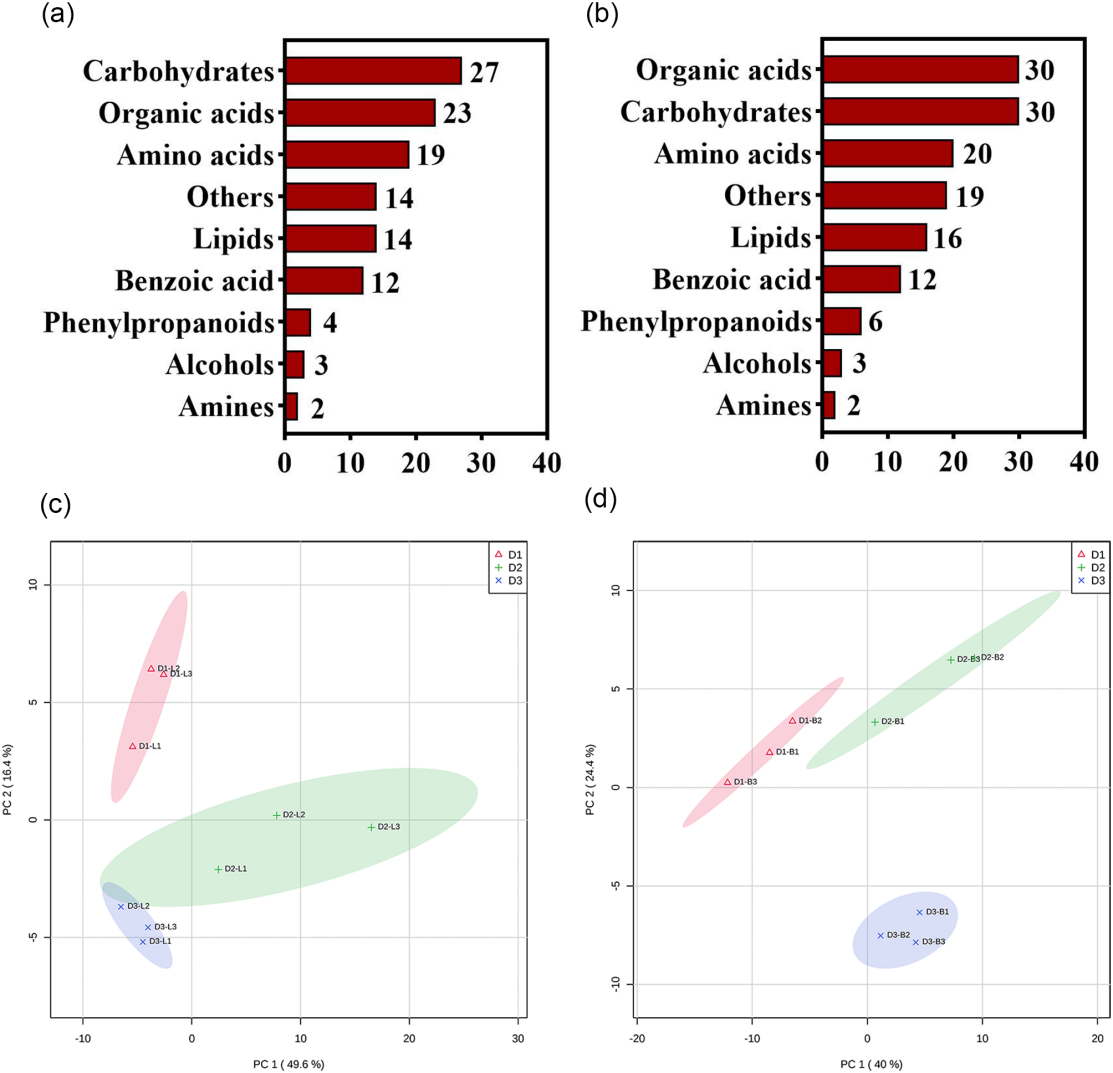
The identified metabolites analysis. Number of different types of metabolites identified in leaves (**a**) and barks (**b**). Principal component analysis (PCA) of the leaves (**c**) and barks (**d**)

To further understand the metabolic changes in leaves and barks of *T. chinensis* in different diameter classes. The primary metabolites in the samples were identified using non-targeted GC-MS metabolomics technology. A total of 118 metabolites were identified in leaves, including 27 carbohydrates, 23 organic acids, 19 amino acids, 14 lipids, 12 benzoic acid, 4 phenylpropanoids, 3 alcohols, 2 amines and 14 others (Fig. 3a). A total of 138 metabolites were divided into 9 classes in barks, including 30 organic acids, 30 carbohydrates, 20 amino acids, 16 lipids, 12 benzoic acid, 6 phenylpropanoids, 3 alcohols, 2 amines and 19 others (Fig. 3b). All samples were subjected to PCA to investigate overall metabolite differences among the different diameter classes (D1, D2, and D3) and intragroup variation in metabolites. PCA results from the three sample groups showed that leaves and barks of *T. chinensis* with different diameter classes were separated, indicating significant metabolic differences (Fig. 3c, d). The results showed that the first principal component (PC1) separated D1, D3 and D2 leaves with 49.6% variance contribution value; D1 was mainly separated by the second principal component (PC2), reaching a value of 16.4%. In the PCA plot of barks, two principal components, PC1 and PC2, were extracted and explained 40.0% and 24.4% of the variability, respectively.

### Differential metabolites between different diameter classes in leaves and barks

**Fig. 4:**
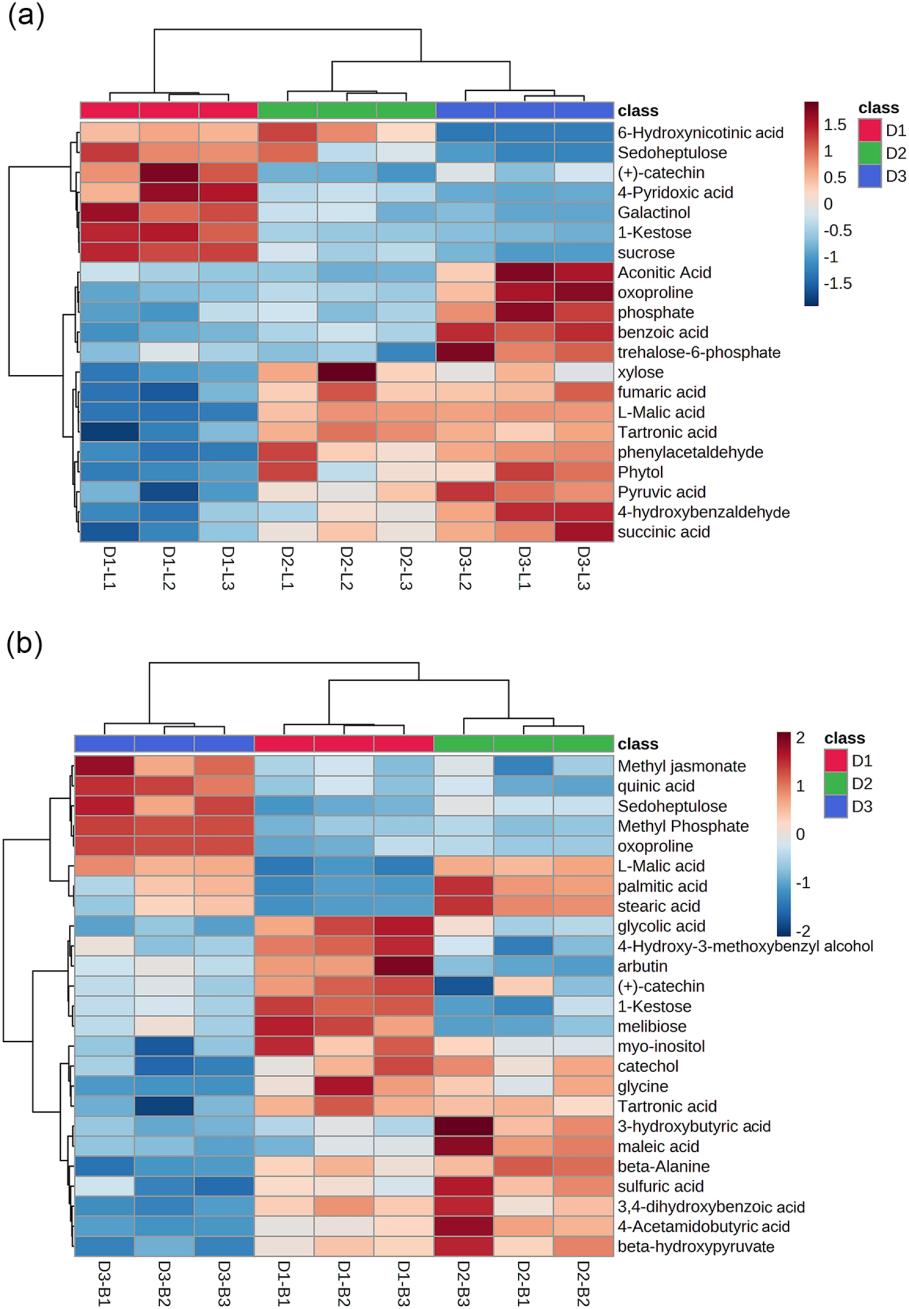
Heatmap of the differential metabolites in leaves (**a**) and barks (**b**)

To identify the differential metabolites between different diameter classes in leaves and barks, we selected the metabolites of variable influence on project values (VIP > 1.0) and *P* value (*P*-value, *P* < 0.05) of one-way ANOVA as the differential metabolites. In three different diameter classes, 21 and 25 different metabolites were screened from leaves and barks, respectively. The main differential metabolites in leaves were mostly organic acids and sugars, and the relative content of most organic acids was higher in D2 and D3 than in D1, such as fumaric acid, L-malic acid, succinic acid and pyruvic acid. The differential metabolism in barks was mostly organic acids and amino acids, and most of the organic acids accumulated more in D2, such as L-malic acid, palmitic acid and stearic acid (Fig. 4).

**Fig. 5:**
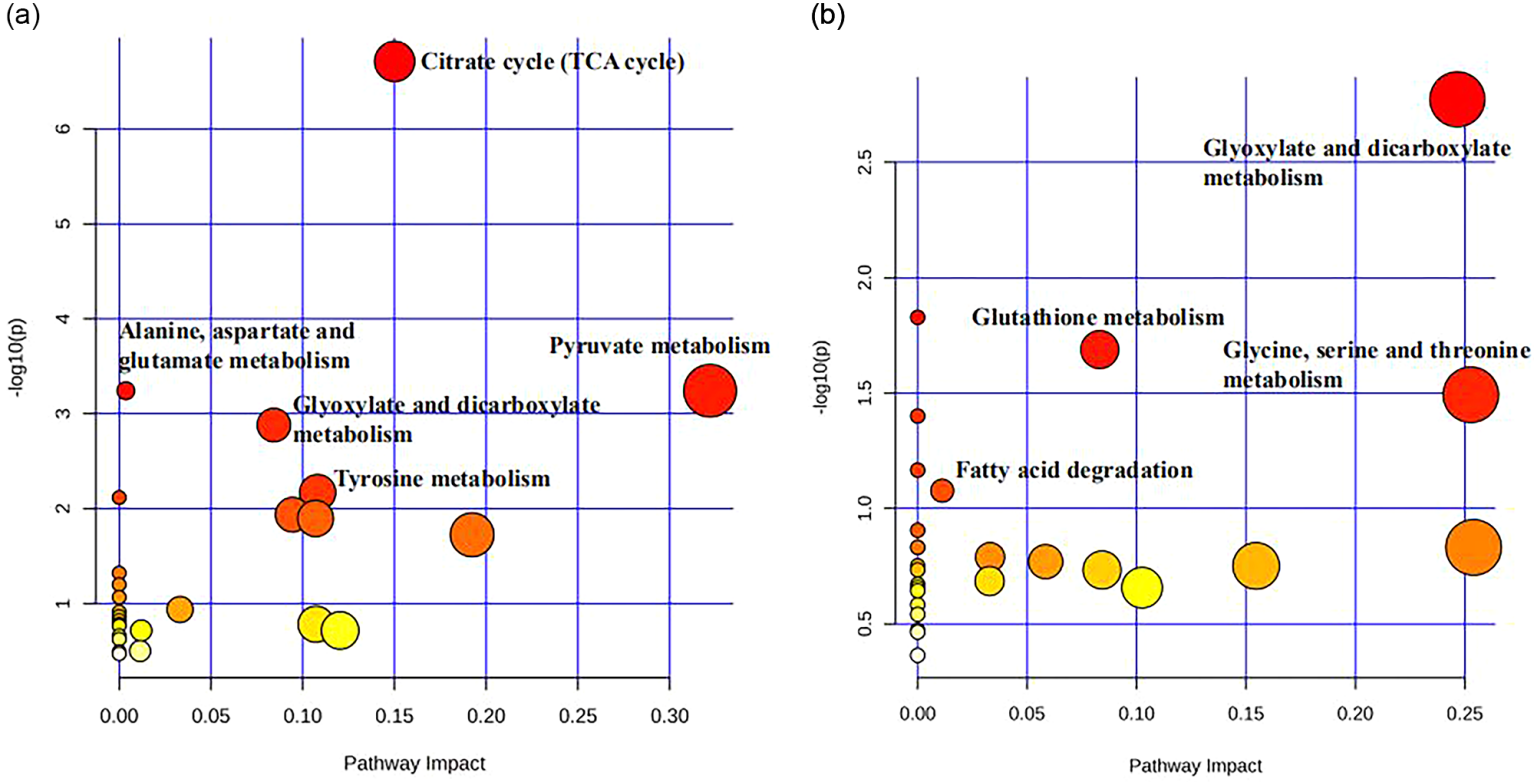
Enriched metabolic pathways of the differential metabolites in leaves (**a**) and barks (**b**). The relevant metabolic pathways in the figure have obtained the appropriate copyright permission of the KEGG image

In addition, we matched all differential metabolites in *T. chinensis* with the KEGG database to obtain information about the pathways involved in metabolites. The metabolic pathway analysis was shown in Fig. 5. The differential metabolites in leaves were mainly enriched in the citrate cycle (TCA cycle), pyruvate metabolism, tyrosine metabolism, alanine, aspartate and glutamate metabolism, and glyoxylate and dicarboxylate metabolism (Fig. 5a). The differential metabolites in barks were annotated and enriched in glyoxylate and dicarboxylate metabolism, glutathione metabolism, and glycine, serine, and threonine metabolism (Fig. 5b).

### Correlation analysis between significantly different factors

**Fig. 6:**
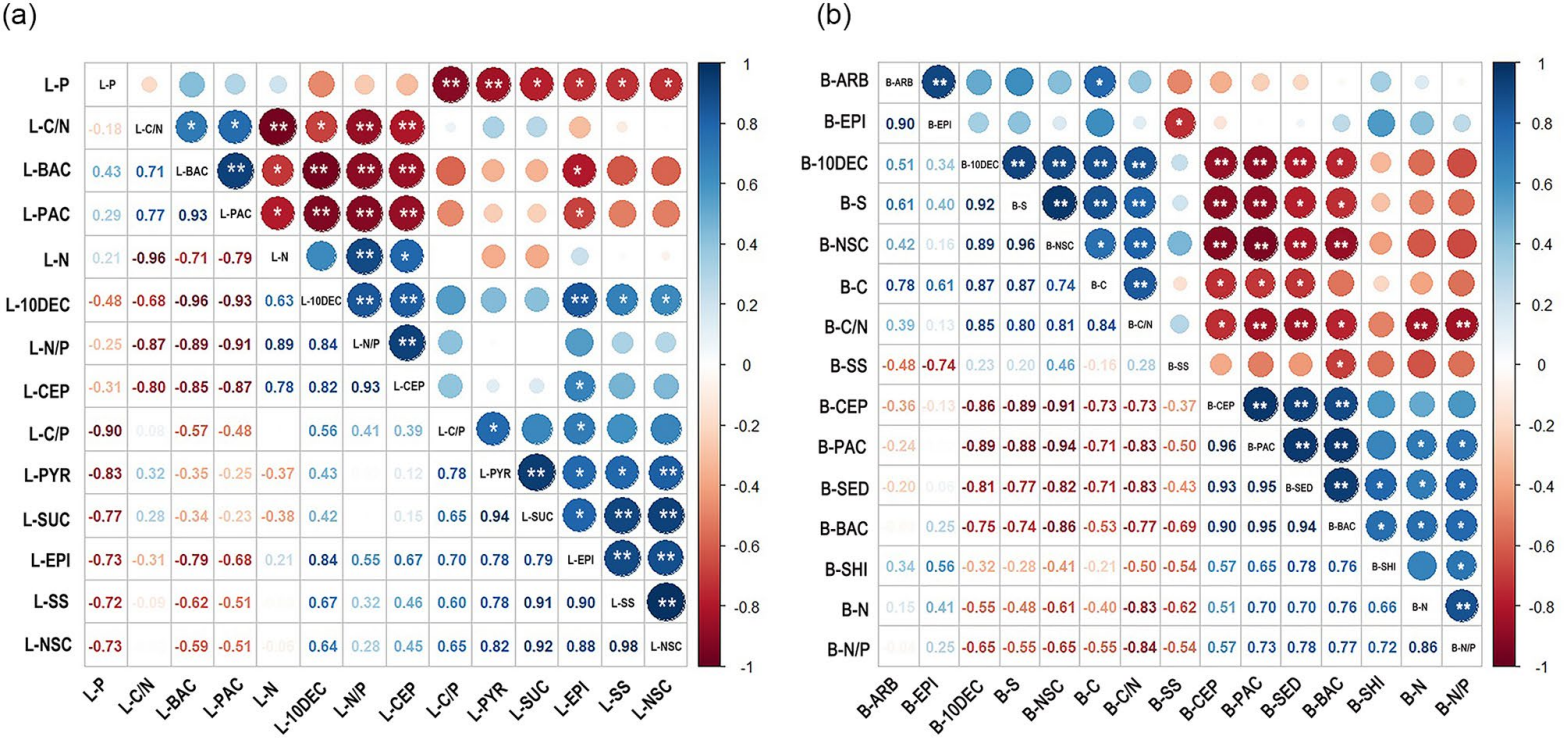
Summary of the correlations (Pearson’s r) of *T. chinensis* in leaves (**a**) and barks (**b**). * indicates significant differences (*P* < 0.05); ** indicates very significant difference (*P* < 0.01). *Abbreviations* C, carbon content; N, nitrogen content; P, phosphorus content; C/N, C:N ratio; N/P, N:P ratio; C/P, C:P ratio; SS, soluble sugar content; S, starch content; NSC, non-structural carbohydrates content; 10DEC, 10-deacetylbaccatin III content; BAC, baccatin III content; CEP, cephalomannine content; PAC, paclitaxel content; PYR, 4-pyridoxic acid content; EPI, epicatechin content; SUC, sucrose content; ARB, arbutin content; SED, sedoheptulose content; SHI, shikimic acid content

The correlation analysis of indicators with significant differences between different diameter classes of *T. chinensis* was performed, and the results are shown in Fig. 6. A total of 44 significant correlations were found, with 24 showing a positive correlation and 20 showing a negative correlation in leaves. In leaves, 4-pyridoxic acid was significantly positively correlated with sucrose, soluble sugars, and non-structural carbohydrates, and negatively correlated with N and P. Epicatechin was significantly negatively correlated with baccatin III, paclitaxel, and negatively correlated with 10-deacetylbaccatin III and cephalomannine. A total of 51 significant correlations, 28 positive correlations and 23 negative correlations were found among the 15 indicators that differed significantly between different diameter classes in the barks. In barks, 10-deacetylbaccatin III showed significant positive correlations with starch, non-structural carbohydrates and C, and negative correlations with shikimic acid and N. Other active substances, including cephalomannine, paclitaxel and baccatin III, showed significant negative correlations with starch, non-structural carbohydrates and C, and positive correlations with N.

## Discussion

The *T. chinensis* exhibits different growth responses in different diameter classes. In our study, the primary metabolites of *T. chinensis* was extensively analyzed. The results showed that the differential metabolites of leaves and barks among different diameter classes were mainly amino acids and organic acids, primarily enriched in the amino acid biosynthesis pathway and energy metabolism pathway. In addition, with increasing diameter class, C and NSCs content decreased, while N content increased. Active substances, such as paclitaxel, accumulated in barks of higher diameter classes.

The results demonstrate a significant impact of diameter class on NSCs content. It has been shown that when the overall carbohydrate supply of plants is sufficient, leaves accumulate a certain amount of NSCs, leading to a downregulation of photosynthesis. Conversely, when the carbohydrate supply is insufficient, demand far exceeds supply, NSCs content in leaves decreases and leaf photosynthetic rate increases [31]. In this study, NSCs content decreased gradually with increasing diameter class, and changes in NSCs content in leaves were mainly influenced by soluble sugars. Soluble sugars participate in plant osmotic regulation, transportation, and signal transduction. Trees preferentially allocate photosynthetic products to soluble sugars to maintain their concentration above a certain threshold [32]. This may be related to the growth season of plants, Yan et al. found that NSCs content in current year needles increased with age during leaf unfolding but significantly decreased with tree age at the end of the growth season [33]. This is consistent with the results of our study. While *T. chinensis* is an evergreen species, its growing season is mainly concentrated in June-July. In this study, the leaves of the current year were collected in September, the fruiting period for *T. chinensis*. In addition, the trend of NSCs content decreasing with the increase of diameter class was also observed in the barks of *T. chinensis*. This is because barks serve as the main NSCs storage organ and tends to store more starch [34]. In summary, our results demonstrate that trees with small diameter classes tend to maintain higher levels of NSCs during the non-growing season to cope with unfavorable conditions and improve their survivability.

In general, higher leaf C content is associated with lower photosynthesis rate and growth rate, and the stronger the defense against unfavorable environment. Conversely, higher leaf N and P content is linked to higher photosynthetic and growth rates and greater resource competitiveness [35, 36]. Our study found that the C content of leaves and barks in *T. chinensis* was higher at D1 and D3, suggesting a stronger defense in the current year leaves of young and old trees. The accumulation of NSCs also confirms this conclusion. Meanwhile, N and P contents in this study all increased with the increase of diameter class, with higher N and P contents in D2 and D3, indicating that resource competitiveness increased with the increase of diameter class. In addition, ecological stoichiometry is related to RNA allocation and cell growth rate, with high growth rates requiring more rRNA for growth [37]. Matzek et al. found that an increase in rRNA content leads to an increase in cellular P content, while C: P and N: P ratios show a decreasing trend during rapid plant growth [38]. In this study, C: P and N: P ratios in leaves were smaller in D1 and D2, indicating faster growth rates during the young and middle ages of *T. chinensis*. Conversely, in order to resist harsh environments, older trees reduce their growth rate, which resulting in relatively high C: P and N: P ratios in leaves [39].

Paclitaxel, 10-deacetylbaccatin III, baccatin III and cephalomannine belong to the paclitaxel group, exhibiting structural similarity with paclitaxel and serve as semi-synthetic precursors for paclitaxel drugs [40, 41]. Previous studies have shown that paclitaxel content in *T. chinensis* is affected by various factors such as species, organs, collection time, and growth environment [3, 24]. In this study, we found that the active components in barks of different diameter classes were significantly higher than those in leaves of the current year, consistent with findings by van Rozendaal et al. [42]. Meanwhile, with the increase of the diameter class, the active substances in the barks of *T. chinensis* showed an increasing trend, except for 10-deacetylbaccatin. Conversely, the leaves showed a decreasing and then increasing trend. This could be attributed to all selected *T. chinensis* in this study were female plants. Young and old trees allocated more energy for defense, accompanied by an increase in secondary metabolites, such as paclitaxel, while medium-sized *T. chinensis* used more energy for reproduction, leading to decreased nutrient growth [43, 44]. In addition, correlation analysis by differential metabolites among different diameter classes revealed significant correlations between various primary metabolites, such as sugars, amino acids and organic acids, and active substances such as paclitaxel. Moreover, differential metabolites associated with active substances in leaves and barks were mainly enriched in the amino acid biosynthetic pathway. Steele et al. reported that D-phenylalanine can effectively increase the content of paclitaxel, presumably due to the molecular structure of D-phenylalanine, which is directly involved in the synthesis of the C-13 side chain of paclitaxel. Additionally, the introduction of D-phenylalanine appears to create a favorable environment for paclitaxel synthesis, promoting cellular metabolic processes and ultimately increasing paclitaxel output [45].

In addition, this study compared metabolic changes during paclitaxel accumulation in leaves and barks of *T. chinensis* of different diameter classes. As the diameter class increased, we observed changes in a series of metabolites including sugars, amino acids and organic acids (Fig. 4). In leaves, pyruvic acid, fumaric acid, L-malic acid and succinic acid accumulated mainly in D2 and D3. Moreover, the TCA cycle and related amino acid metabolic pathways including alanine, aspartate and glutamate metabolism were also enhanced. The TCA cycle involves the biosynthesis of fumaric acid, isocitrate, and succinic acid, many of which are precursors for amino acid biosynthesis [46]. Glutamate serves as a substrate for glutamate synthase, which synthesizes N compounds such as proteins and nucleic acids [47, 48]. This indicated that N metabolism was enhanced in the leaves of middle-aged and old trees compared to young trees. In addition, L-malic acid, palmitic acid and stearic acid in barks were found to accumulate significantly in D2 and D3. The upregulation of organic acids promotes sugar metabolism and the TCA cycle, providing energy to initiate and maintain the antioxidant system [49, 50]. Pathway analysis showed that differential metabolites were mainly enriched in glyoxylate and dicarboxylate metabolism, glutathione metabolism, and glycine, serine, and threonine metabolism. Glyoxylate and dicarboxylate metabolism regulate carbohydrate metabolism and enhance plant tolerance [51, 52]. In summary, as the diameter class increases, it is regulated by metabolites such as amino acids and organic acids, ultimately resulting in enhanced nitrogen metabolism in plants at higher diameter classes.

## Conclusions

As a valued medicinal plant, the growth age of *T. chinensis* is a crucial factor that affects its medicinal value. In this study, we compared the variations in NSCs, nutrients, active substances and primary metabolites in barks and leaves of the current year from different diameter classes. The results showed that smaller diameter classes had higher C and NSCs content in both leaves and barks, thereby enhancing defense capabilities. N content in leaves and barks increased with diameter class and enhanced TCA cycle to support amino acid conversion for the synthesis of active substances, such as paclitaxel. In conclusion, our results confirm that different diameter classes of *T. chinensis* affect the accumulation of active ingredients through flexible allocation of carbon and nitrogen metabolism, providing a basis for the harvesting and utilization of *T. chinensis*. In addition, our results provide inspiration for the future by regulating carbon and nitrogen metabolism, thereby increasing the accumulation of paclitaxel.

## Data Availability

The datasets used and/or analyzed during the current study available from the corresponding author on reasonable request.
